# Long-term monitoring of gastric mucosa-associated lymphoid tissue lymphoma in patients with extra copies of the *MALT1* gene

**DOI:** 10.1038/s41598-024-55663-9

**Published:** 2024-02-29

**Authors:** Masaya Iwamuro, Ryuta Takenaka, Koji Miyahara, Shotaro Okanoue, Masao Yoshioka, Chihiro Sakaguchi, Kumiko Yamamoto, Yoshinari Kawai, Tatsuya Toyokawa, Takehiro Tanaka, Motoyuki Otsuka

**Affiliations:** 1 Department of Gastroenterology and Hepatology, Okayama University Graduate School of Medicine, Dentistry, and Pharmaceutical Sciences, 2-5-1 Shikata-cho, Kita-ku, Okayama, 700-8558 Japan; 2 Department of Internal Medicine, Tsuyama Chuo Hospital, Tsuyama, 708‑0841 Japan; 3 Department of Internal Medicine, Hiroshima City Hospital, Hiroshima, 730-8518 Japan; 4 Department of Gastroenterology, Mitoyo General Hospital, Kanonji, 769‑1695 Japan; 5 Department of Internal Medicine, Okayama Saiseikai General Hospital, Okayama, 700‑8511 Japan; 6 Department of Gastroenterology, Shikoku Cancer Center, Matsuyama, 791-0280 Japan; 7 Department of Gastroenterology, Kagawa Prefectural Central Hospital, Takamatsu, 760‑8557 Japan; 8 Department of Gastroenterology, Onomichi Municipal Hospital, Onomichi, 722‑8503 Japan; 9 Department of Gastroenterology, Fukuyama Medical Center, Fukuyama, 720‑8520 Japan; 10 Department of Pathology, Okayama University Hospital, Okayama, 700-8558 Japan

**Keywords:** Extranodal marginal zone lymphoma of mucosa-associated lymphoid tissue, Gastric neoplasms, Esophagogastroduodenoscopy, t(11;18) translocation, Trisomy 18, Cancer, Gastrointestinal cancer, Haematological cancer

## Abstract

The objective of this study was to clarify the long-term prognosis of patients with gastric mucosa-associated lymphoid tissue (MALT) lymphoma with additional copies of *MALT1*. In this multicenter retrospective study, we enrolled 145 patients with gastric MALT lymphoma who underwent fluorescence in situ hybridization (FISH) analysis to detect t(11;18) translocation. The patient cohort was divided into three groups: Group A (n = 87), comprising individuals devoid of the t(11;18) translocation or extra *MALT1* copies; Group B (n = 27), encompassing patients characterized by the presence of the t(11;18) translocation; and Group C (n = 31), including patients with extra *MALT1* copies. The clinical outcomes in each cohort were collected. Over the course of a mean follow-up of 8.5 ± 4.2 years, one patient died of progressive MALT lymphoma, while 15 patients died due to etiologies unrelated to lymphoma. The progression or relapse of MALT lymphoma was observed in 11 patients: three in Group A, two in Group B, and six in Group C. In Groups A, B, and C, the 10-year overall survival rates were 82.5%, 93.8%, and 86.4%, respectively, and the 10-year event-free survival rates were 96.1%, 96.0%, and 82.9%, respectively. The event-free survival rate in Group C was significantly lower than that in Group A. However, no differences were observed in the 10-year event-free survival rates among individuals limited to stage I or II_1_ disease (equivalent to excluding patients with stage IV disease in this study, as there were no patients with stage II_2_), with rates of 98.6%, 95.8%, and 92.3% for Groups A, B, and C, respectively. In conclusion, the presence of extra copies of *MALT1* was identified as an inferior prognostic determinant of event-free survival. Consequently, trisomy/tetrasomy 18 may serve as an indicator of progression and refractoriness to therapeutic intervention in patients with gastric MALT lymphoma, particularly stage IV gastric MALT lymphoma.

## Introduction

Trisomy and tetrasomy 18 are genetic aberrations characterized by the presence of one or two additional copies of chromosome 18 within a subset or in the entirety of cellular components. This genomic anomaly is a prevalent karyotypic irregularity in extranodal marginal zone lymphoma of mucosa-associated lymphoid tissue (MALT lymphoma), which is a type of lymphoma that originates from mucosa-associated lymphoid tissue in various organs, including the stomach, lungs, eyes, and thyroid^1–5^. Trisomy and tetrasomy 18 are typically diagnosed by fluorescence in situ hybridization (FISH) analysis employing centromere-specific probes targeted to chromosome 18 or, alternatively, via array comparative genomic hybridization or cytogenetic analysis (G-banding). However, an alternative method of identification exists, which entails the recognition of surplus *MALT1* gene copies using a FISH assay devised for probing the t(11;18)(q21;q21)/*BIRC3-MALT1* translocation because *MALT1* is located on chromosome 18q21.

In our previous investigation, we examined 146 patients with MALT lymphoma of the stomach who underwent FISH analysis for t(11, 18) translocation^6^. The patients were stratified into patients without t(11;18) translocation or extra copies of *MALT1* (Group A), those with t(11;18) translocation (Group B), and those with extra copies of *MALT1* (Group C). We demonstrated that Group C patients had a prevalence of *H. pylori* infection, and the response rate to *H. pylori* eradication treatment was comparable to that of Group A patients. Although the log-rank test indicated no significant difference between Groups A and C, the event-free survival of patients in Group C appeared to be inferior to that of patients in Groups A and B. These results indicate that trisomy or tetrasomy 18 may be a marker of poor prognosis and resistance to treatment in patients with gastric MALT lymphoma. However, the importance of trisomy and tetrasomy 18 in gastric MALT lymphoma is not fully understood because of the lack of long-term patient monitoring focused on genetic abnormalities. Therefore, in this study, we extended our investigation by tracking the same cohort of patients for an additional period exceeding 5 years to analyze the long-term prognosis of patients with gastric MALT lymphoma harboring trisomy/tetrasomy 18.

## Materials and methods

As described above, this study involved tracking the same group of patients as in our previous investigation for an additional 5 years and analyzing their long-term prognosis^6^. A thorough re-examination of the medical records of the cohort revealed that in one patient without t(11;18) translocation or extra copies of *MALT1*, the initial diagnosis was diffuse large B-cell lymphoma (DLBCL) rather than MALT lymphoma. Hence, we excluded this patient from the analysis. Ultimately, we analyzed 145 patients who were diagnosed with gastric MALT lymphoma between October 1997 and November 2015. All patients had undergone FISH analysis targeting the t(11;18)(q21;q21)/*BIRC3-MALT1* translocation.

In accordance with the FISH results for the t(11;18) translocation, the patients were stratified into the following three cohorts: (1) Group A, encompassing patients devoid of the t(11;18)(q21;q21)/*BIRC3-MALT1* translocation or any additional copies of *MALT1*; (2) Group B, comprising patients exhibiting the t(11;18) translocation; and (3) Group C, comprising patients characterized by an excess of *MALT1* copies. The follow-up period was defined as the time from the initial lymphoma diagnosis to mortality stemming from any causal factor or culminating in the patient’s last hospital visit. Disease-specific survival was defined as the probability of patients with MALT lymphoma surviving during the observation period, starting from diagnosis until death from MALT lymphoma. Event-free survival was quantified, commencing from the moment of diagnosis and persisting until documented progression/relapse, death from the primary disease, or commencement of the second treatment for any reason. For the comparisons of two groups, statistical assessments, including t‑tests, χ^2^ tests, and F‑tests, were performed using JMP 16.0.0 software (SAS Institute, Cary, NC, United States).

Cumulative probabilities of event-free survival were derived via Kaplan–Meier analysis with subsequent log-rank tests using JMP 16.0.0 software. A significance threshold of P < 0.05 was upheld as a hallmark signifying statistically salient distinctions between the cohorts under scrutiny. This study was approved by the Ethical Committee of Okayama University Hospital and adhered to the Declaration of Helsinki. The requirement for written informed consent was waived by the ethics committees of Okayama University Hospital and the participating institutions due to the retrospective nature of the study and the use of anonymous clinical data for analysis.

## Results

As mentioned above, the present investigation was directed toward the same cohort of patients^6^, with the sole exclusion being one patient with diffuse large B-cell lymphoma, over a prolonged period of observation. Consequently, the patient demographics remained almost identical to those in our previous study. The cohort consisted of 76 women and 69 men, with a mean age of 65.5 ± 12.6 years. The baseline characteristics of the patient subgroups devoid of the t(11;18) translocation or additional *MALT1* copies (Group A), those possessing the t(11;18)(q21;q21) translocation (Group B), and those with extra copies of *MALT1* (Group C) are shown in Table 1. The abnormalities of chromosome 18 in Group C, suspected from extra copies of the *MALT1* gene, included trisomy (n = 27), both trisomy and tetrasomy (n = 2), tetrasomy (n = 1), and both trisomy and tetraploidy (n = 1). Comparisons between Groups A, B, and C revealed no significant differences in sex or age, whereas the infection rate of *H. pylori* in Group B (2/25, 7.4%) was significantly lower than that in Group A (65/87, 74.7%). Despite no statistically significant differences among the groups regarding lymphoma stage, Group C had a greater proportion of patients with stage IV disease (4/31, 12.9%) than Groups A (5/87, 5.7%) and B (2/27, 7.4%).

**Table 1:** Clinical backgrounds of the study subjects, n (%).

	Total	Group A: no chromosome aberration	Group B: t(11;18) positive	Group C: extra copies of *MALT1*	Group A vs. B, p value	Group A vs. C, p value
N	145	87	27	31		
Sex					0.125	0.835
Male	69	39	17	13		
Female	76	48	10	18		
Age (mean ± SD, y)	65.5 ± 12.6	66.1 ± 12.2	64.2 ± 11.2	65.0 ± 14.9	0.501	0.684
Stage (Lugano system)					0.669^a^	0.240^a^
I	131	81	24	26		
II1	3	1	1	1		
II2	0	0	0	0		
IV	11	5	2	4		
*H. pylori*					< 0.001	0.454
Positive	88	65	2	21		
Negative	57	22	25	10		
Treatment					< 0.001^b^	0.271^b^
Eradication alone	81	60	3	18		
RT alone	23	8	15	0		
Chemotherapy alone	8	3	2	3		
RT and chemotherapy	2	1	0	1		
Eradication and RT	21	8	5	8		
Eradication and chemotherapy	2	1	0	1		
Eradication, RT, and chemotherapy	2	1	1	0		
None	6	5	1	0		
Outcome					0.295^c^	0.756^c^
Live without disease	105	64	20	21		
Live with disease	20	9	4	7		
Live, unknown disease status	4	2	2	0		
Dead by other cause	15	11	1	3		
Dead by MALT lymphoma	1	1	0	0		
Progression or recurrence of MALT lymphoma	11	3	2	6		
Follow-up period (mean ± SD, y)	8.5 ± 4.2	8.2 ± 4.0	9.7 ± 4.8	8.2 ± 3.9		

*RT* radiotherapy.

^a^Stages I & II_1_ vs. IV.

^b^Eradication alone vs. other.

^c^Alive vs. dead.

The initial treatment strategies included eradication, radiotherapy, chemotherapy, or a combination of these therapies, but some patients were not receiving specific treatments (Table 1). The majority of patients in Groups A (60/87, 69.0%) and C (18/31, 58.1%) received *H. pylori* eradication therapy alone, whereas only 3 of 27 patients (11.1%) in Group B underwent *H. pylori* eradication treatment alone. The mean follow-up period was 8.5 ± 4.2 years in the entire group, 8.2 ± 4.0 years in Group A, 9.7 ± 4.8 years in Group B, and 8.2 ± 3.9 years in Group C. Overall, 16 patients died during the observation period. One patient died due to MALT lymphoma progression, and the causes of death in the other patients were as follows: pneumonia (n = 4), malignant melanoma (n = 1), lung cancer (n = 1), pancreatic cancer (n = 1), liposarcoma (n = 1), influenza virus infection (n = 1), decompensated liver cirrhosis (n = 1), aortic dissection (n = 1), drowning (n = 1), chronic subdural hematoma (n = 1), senescence (n = 1), and undetermined causes (n = 1). Among the remaining patients who were alive at their most recent visit to the medical facility, 105 exhibited no evidence of MALT lymphoma, while 20 had lymphoma. However, the disease status of four patients remained undetermined.

The progression or recurrence of MALT lymphoma was observed in 11 patients, comprising three patients in Group A, two patients in Group B, and six patients in Group C (Table 2). Two patients in Group C who experienced recurrence (patients no. 7 and 8) were previously described in case reports^7,8^. Three patients (patients no. 1, 4, and 5) experienced a relapse of gastric MALT lymphoma 3.2, 6.3, and 11.3 years after radiotherapy, respectively. In two patients, the disease promptly reverted to complete remission without therapeutic intervention, while the remaining patient refused any therapeutic intervention for relapsed lymphoma due to the absence of symptomatic manifestations. A patient diagnosed with stage IV lymphoma, characterized by involvement of the stomach, ileum, colon, rectum, and systemic lymph nodes (patient no. 2), experienced disease progression 1 year after the initiation of chemotherapy and ultimately succumbed due to intestinal perforation. One patient with stomach and lung involvement (patient no. 3) underwent eradication of *H. pylori* and radiotherapy to the stomach, followed by chemotherapy. An increase in gastric lesions was observed 4.7 years after diagnosis, and transformation to DLBCL was confirmed by endoscopic biopsy. Although rituximab monotherapy was initiated, the patient was transferred because of a femoral fracture, and the subsequent outcomes remain unknown. In one patient (patient no. 6), eradication therapy was administered despite *H. pylori* negativity; however, the gastric MALT lymphoma failed to regress, leading to the initiation of radiotherapy. Nevertheless, 2.1 years later, ileal and pharyngeal MALT lymphoma lesions developed, prompting the initiation of rituximab monotherapy. A patient diagnosed with stage IV MALT lymphoma (patient no. 9) underwent antibiotic therapy despite testing negative for *H. pylori* and was subsequently maintained under active surveillance owing to asymptomatic presentation. Intra-abdominal lymph node enlargement worsened 1.4 years later, leading to a hydroureter and prompting a transfer to another facility where chemotherapy was initiated. The remaining two patients with stage IV MALT lymphoma (patients no. 10 and 11) underwent chemotherapy and experienced disease relapse after 1.8 and 5.2 years, respectively. One patient died of influenza pneumonia during chemotherapy.

**Table 2:** Patients who experienced relapse or progression of MALT lymphoma.

Case no.	Age (y)	Sex	Chromosome aberration	Stage	Involved organs	Treatment	Follow-up period (y)	Outcome	Time to events (y)	Events
1	73	M	No chromosome aberration	I	Stomach	RT alone	9.4	Live without disease	3.2	Relapsed but spontaneously reverted to CR
2	83	F	No chromosome aberration	IV	Stomach, ileum, colon, rectum, systemic lymph nodes	Chemotherapy alone	1.4	Dead by MALT lymphoma	1.0	Progression of lymphoma
3	80	F	No chromosome aberration	IV	Stomach, lung	Eradication, RT, and chemotherapy	4.8	Live with disease	4.7	Transformation to DLBCL
4	78	M	t(11;18) positive	I	Stomach	RT alone	6.3	Live with disease	3.5	Relapsed
5	62	M	t(11;18) positive	I	Stomach	Eradication and RT	17.3	Live with disease	11.3	Relapsed but spontaneously reverted to CR
6	44	M	Extra copies of *MALT1*	I	Stomach	Eradication and RT	7.7	Live with disease	2.1	Progression of lymphoma
7	35	F	Extra copies of *MALT1*	I	Stomach	Eradication and RT	12.0	Live without disease	2.4	Relapsed
8	39	M	Extra copies of *MALT1*	I	Stomach	Eradication alone	13.8	Live with disease	13.7	Relapsed
9	67	M	Extra copies of *MALT1*	IV	Stomach, kidney, intraabdominal lymph nodes	Eradication alone	2.2	Live with disease	1.4	Progression of lymphoma
10	79	M	Extra copies of *MALT1*	IV	Stomach, spleen, systemic lymph nodes	Chemotherapy alone	2.0	Dead by other cause	1.8	Relapsed
11	57	F	Extra copies of *MALT1*	IV	Stomach, lung, systemic lymph nodes	Chemotherapy alone	7.2	Live with disease	5.2	Relapsed

*RT* radiotherapy, *CR* complete response, *DLBCL* diffuse large B-cell lymphoma.

**Figure 1:**
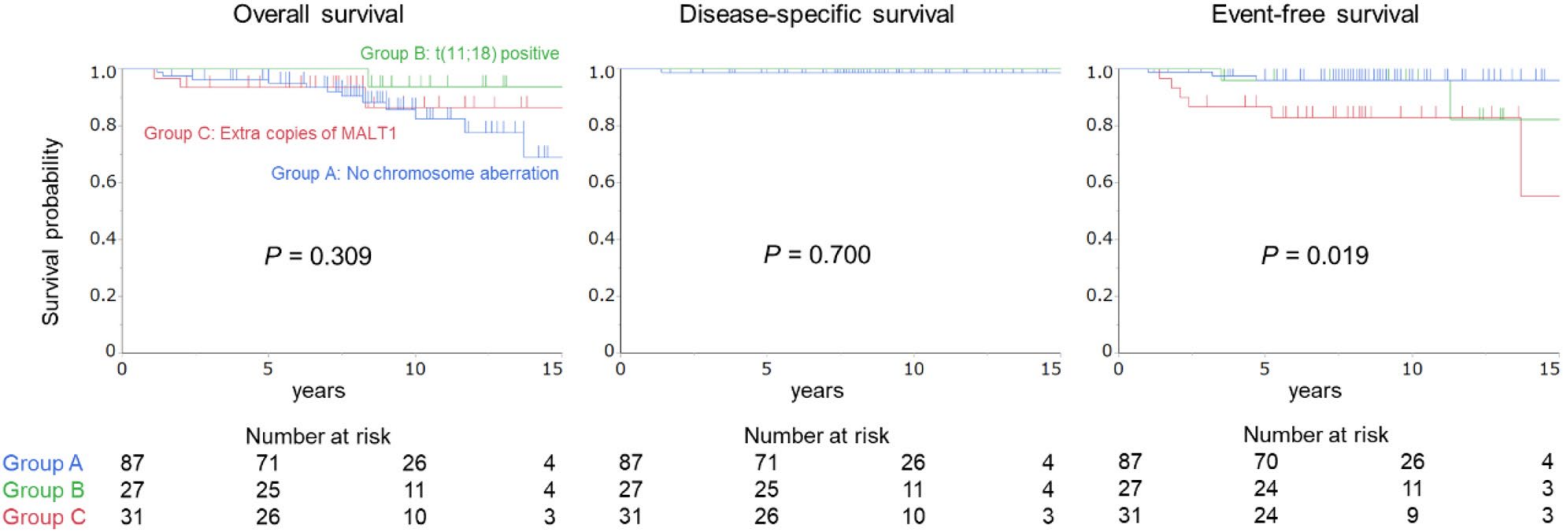
Cumulative survival probabilities for the three groups. The overall and disease-specific survival rates did not differ significantly between the groups; the event-free survival in Group C patients was significantly lower than that of patients in the other two groups.

**Figure 2:**
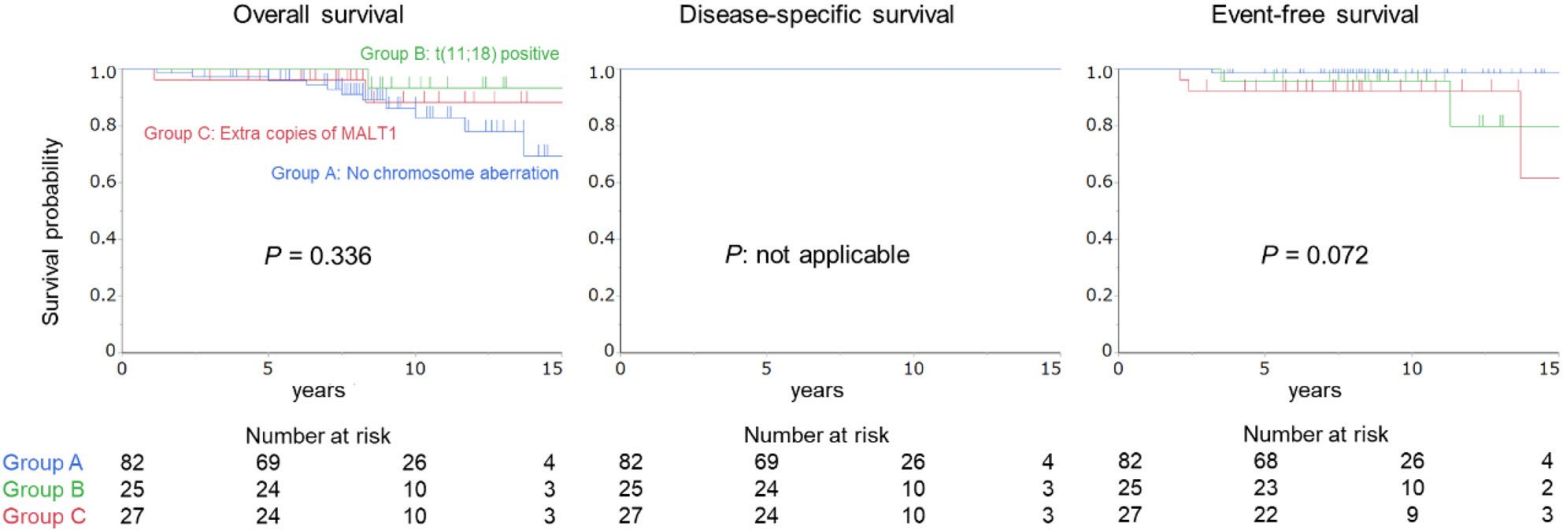
Cumulative survival probabilities for patients with stage I or II_1_ disease. The overall, disease specific, and event-free survival rates were not significantly different between the three groups.

Figure 1 illustrates the Kaplan–Meier analysis outcomes pertaining to the cumulative probabilities of overall, disease specific, and event-free survival across the three cohorts, wherein events were defined as instances of lymphoma progression or recurrence. The 10-year overall survival rates were 82.5% in Group A, 93.8% in Group B, and 86.4% in Group C. Given the solitary instance of lymphoma-related mortality within Group A, the 10-year disease-specific survival rate reached 98.8% for this particular cohort, whereas patients in Groups B and C achieved a 100% survival rate. Moreover, the 10-year event-free survival rates were 96.1% and 96.0% in Groups A and B, respectively, whereas that in Group C was 82.9%. The event-free survival in Group C was significantly lower than that in Group A. Generally, primary gastric MALT lymphoma comprises patients with stage I or II_1_ disease. Subsequently, we conducted an analysis limited to stage I and II_1_ patients (Fig. 2). This analysis revealed no statistically significant differences in event-free survival among the three groups. The 10-year event-free survival rates of patients with stage I or II_1_ disease (excluding patients with stage IV disease) were 98.6%, 95.8%, and 92.3% in Groups A, B, and C, respectively.

## Discussion

This study investigated the long-term outcomes of patients with gastric MALT lymphoma based on the presence of chromosomal aberrations and revealed that progression or recurrence occurs more frequently in patients with extra copies of *MALT1* (Group C). Patients with stage I or II_1_ disease are classified as having primary gastric MALT lymphoma. In contrast, patients with stage IV disease, indicating lesions beyond the abdominal cavity, are classified as having systemic lymphoma, and management falls under hematologists rather than gastroenterologists. In this study, there were no patients with stage II_2_ disease. Therefore, we conducted additional analyses limited to patients with stage I or II_1_ disease (excluding patients with stage IV disease). The subanalysis results focusing on primary gastric MALT lymphoma patients showed no differences in progression-free survival. This can be attributed to the relatively higher prevalence of patients with stage IV disease in Group C. Notably, in Group C, three of four patients (75.0%) who presented with stage IV disease experienced either progression or recurrence, in contrast to the occurrence rates of 40.0% (2/5 patients) in Group A and 0% (0/2 patients) in Group B. Conversely, disease progression or recurrence is rare in limited-stage gastric MALT lymphoma patients (stage I or II_1_), with a 10-year event-free survival rate exceeding 90% across all cohorts. These findings suggest that vigilant monitoring for disease progression or recurrence is warranted, especially for individuals with trisomy/tetrasomy 18 presenting at stage IV.

The clinical importance of trisomy/tetrasomy 18 in gastric MALT lymphomas remains unclear. Krugmann et al. analyzed 29 patients with surgically excised gastric MALT lymphoma or DLBCL^9^. Over a median observational period of 56 months, an increase in the copy number of the *MALT1* locus exhibited a significant association with diminished disease-specific survival, although there was no observable disparity in overall or event-free survival. Nevertheless, the limited sample size rendered the disease-specific survival outcome nonsignificant according to the Cox regression analysis. Nakamura et al. investigated the occurrence of chromosomal translocations and variations in the copy numbers of *MALT1*, *IGH*, and *FOXP1* in 90 patients with gastric MALT lymphoma^10^. Over a mean follow-up interval of 65 months, a significant predilection for lymphoma recurrence or disease relapse emerged in patients exhibiting increased copies of *MALT1* through multivariate analysis in comparison to those without. The findings of the current study reaffirm that excess *MALT1* is associated with an elevated risk of disease progression or relapse in patients with gastric MALT lymphoma. Notably, this conclusion is derived from an extensive dataset, including the largest reported patient cohort to date (n = 145) and an extended mean follow-up duration of 8.5 ± 4.2 years.

It is noteworthy that while the majority of patients experienced disease progression or relapse within a 5-year timeframe (Table 2), two patients with gastric MALT lymphoma experienced a relapse period exceeding a decade after their initial diagnosis. Nakamura et al. reported that, with a median follow-up duration of 5.5 years among 323 respondents who demonstrated pathological remission of MALT lymphoma, 3.1% experienced lymphoma relapse^11^. One patient exhibited macroscopic local relapse of MALT lymphoma 10.9 years after achieving complete remission. Cases of recurrence occurring more than 10 years after complete remission (CR) have also been reported in other studies^12^. These findings indicate that despite the infrequent occurrence of recurrence over an extended temporal span, endoscopic surveillance may be required beyond a decade in patients with gastric MALT lymphoma even after complete remission.

In the present study, which encompassed an extended period of observation, mortality directly linked to MALT lymphoma was observed in only one patient in Group A. Consequently, the 10-year disease-specific survival rate in Group A was 98.8%, whereas both Groups B and C exhibited a 100% 10-year disease-specific survival rate. Furthermore, in the subset of patients with localized stage (stage I or II_1_) gastric MALT lymphoma, the 10-year event-free survival rates were 98.6%, 95.8%, and 92.3% in Groups A, B, and C, respectively. These findings underscore the favorable long-term prognosis associated with gastric MALT lymphoma^13–16^. Moreover, although not statistically significant, it is noteworthy that Group A exhibited a greater number of deaths, particularly due to nonlymphoma-related causes (n = 11). However, these incidents were unrelated to lymphoma or its treatment, which we attributed to incidental factors.

This study has several limitations that warrant discussion. First, we did not perform a comprehensive assessment of chromosomal alterations. MALT lymphomas encompass a spectrum of chromosomal aberrations, including trisomies involving chromosomes 3, 7, and 12, as well as translocations such as t(14;18) and t(3;14)^17,18^. Consequently, Group A could have exhibited heterogeneity, potentially harboring patients with chromosomal abnormalities other than t(11;18) translocation and trisomy/tetrasomy 18. Second, the study exclusively enrolled patients who underwent FISH for t(11;18) translocations. FISH analysis may have been waived in patients in which *H. pylori* was promptly eradicated upon the diagnosis of MALT lymphoma, resulting in lymphoma regression. Consequently, there is a possibility of selection bias within this study. Third, owing to the retrospective and multicenter nature of this study, the treatment modalities administered to each patient may have exhibited substantial variability contingent on the predilections of the attending physicians and the accessibility of chemotherapy and/or radiotherapy at individual institutions. Conversely, the findings derived from this study, based on real-world clinical data encompassing FISH, can be readily translated into clinical practice.

In summary, the presence of additional copies of *MALT1* has emerged as an adverse prognostic determinant of event-free survival. Consequently, trisomy/tetrasomy 18 may serve as an indicator of progression and resistance to treatment in patients with gastric MALT lymphoma, particularly in those with stage IV disease. More active follow-up and shortening of the intervals between examinations such as esophagogastroduodenoscopy and CT scans may be necessary for these patients. Although further investigation is imperative to determine the molecular intricacies governing the involvement of trisomy/tetrasomy 18, the present study involves the longest observational period ever reported, with detailed information on chromosomal alterations in gastric MALT lymphoma. This extensive dataset facilitated the estimation of the 10-year event-free survival in each cohort. We believe that the results of our long-term observational study will help gastroenterologists outline possible outcomes for patients with this disease.

## Data Availability

The datasets generated and analyzed in the current study are available from the corresponding author upon reasonable request.

## Abbreviations

CR: Complete remission
FISH: Fluorescence in situ hybridization
MALT lymphoma: Extranodal marginal zone lymphoma of mucosa-associated lymphoid tissue
